# The efficacy of NMDA receptor antagonists in neuropsychiatric and non-motor symptoms of Parkinson’s disease: a systematic review

**DOI:** 10.1590/1980-5764-DN-2026-0496

**Published:** 2026-11-02

**Authors:** Jan Arel Solomon Orlino, Ma. Lourdes Corrales-Joson

**Affiliations:** 1 University of Santo Tomas Hospital, Department of Neuroscience and Behavioral Medicine, Manila, Philippines.; 2 University of Santo Tomas, Faculty of Medicine and Surgery, Department of Neuroscience and Behavioral Medicine, Manila, Philippines.

**Keywords:** Parkinson Disease, Memantine, Amantadine, Systematic Review, Doença de Parkinson, Memantina, Amantadina, Revisão Sistemática

## Abstract

Parkinson’s disease (PD) involves significant non-motor symptoms (NMS) linked to glutamate dysregulation.

**Objective:**

This review evaluates the efficacy of N-methyl-D-aspartate (NMDA) receptor antagonists (memantine and amantadine) for treating NMS.

**Methods:**

A search was conducted in the databases PubMed and Cochrane Library (January 2000–September 2025) for controlled trials focusing on NMS in idiopathic PD. Two reviewers assessed bias using Cochrane RoB 2.

**Results:**

Of 529 records, ten studies were included. Most exhibited a high risk of bias and heterogeneity. Memantine results were mixed: improvements in attention and behavioral symptoms were observed in PD dementia, but no benefit or worsened memory occurred in mild cognitive impairment. Memantine was beneficial for REM sleep behavior disorder. Extended-release amantadine improved sleepiness and mood but increased hallucinations and cognitive impairment.

**Conclusion:**

Evidence is limited by methodological flaws. While memantine offers selective benefits for PD dementia, amantadine carries notable cognitive risks. High-quality trials are essential to clarify therapeutic roles.

## RESUMO.

A doença de Parkinson (DP) envolve sintomas não motores (SNM) significativos associados à desregulação do glutamato.

**Objetivo:**

Esta revisão avalia a eficácia dos antagonistas do receptor N-metil-D-aspartato (NMDA) (memantina e amantadina) no tratamento dos SNM.

**Métodos:**

Foi realizada uma busca nas bases de dados PubMed e Cochrane Library (janeiro de 2000 a setembro de 2025) por ensaios clínicos controlados com foco nos SNM da DP idiopática. Dois revisores avaliaram o viés utilizando o Cochrane RoB 2.

**Resultados:**

Dos 529 registros, dez estudos foram incluídos. A maioria apresentou alto risco de viés e heterogeneidade. Os resultados da memantina foram mistos: melhorias na atenção e nos sintomas comportamentais foram observadas na demência associada à DP, mas não houve benefício ou ocorreu piora da memória no comprometimento cognitivo leve. A memantina foi benéfica para o distúrbio comportamental do sono REM. A amantadina de liberação prolongada melhorou a sonolência e o humor, mas aumentou as alucinações e o comprometimento cognitivo.

**Conclusão:**

As evidências são limitadas por falhas metodológicas. Embora a memantina ofereça benefícios seletivos para a demência associada à DP, a amantadina acarreta riscos cognitivos notáveis. Ensaios clínicos de alta qualidade são essenciais para esclarecer os papéis terapêuticos.

## INTRODUCTION

 Parkinson’s disease (PD) is a progressive neurodegenerative disorder characterized by the cardinal motor features of parkinsonism — bradykinesia, tremor, rigidity, and postural instability — resulting from the selective loss of dopaminergic neurons in the substantia nigra pars compacta and the intracellular accumulation of alpha-synuclein in Lewy bodies^1^. 

 The prevalence of PD is reported to be within the range of 1–2 per 1,000 in the general population. Notably, the prevalence rises substantially with advancing age, approximating 1% in those over 60 years^2^. 

 The etiology of PD remains largely idiopathic. While genetic factors play a role, their contribution is variable, with monogenic forms accounting for a small percentage of cases. Overall, genetic factors are estimated to contribute to 5–10% of cases of PD. Environmental factors are also implicated in PD risk^2^. 

 PD extends beyond motor symptoms, with non-motor symptoms (NMS) significantly impacting patient well-being. NMS, including hyposmia, REM sleep behavior disorder, and autonomic dysfunction, often precede or accompany motor deficits. Cognitive decline and psychosis manifest later in the disease course. Neuropathological evidence suggests a caudal-to-rostral progression of pathology, correlating with the emergence of NMS. Untreated NMS contribute to decreased quality of life, increased morbidity, and potential mortality in PD patients^3^. 

 Glutamate, the primary excitatory neurotransmitter in the central nervous system, is essential for neuronal function^4^. Dysregulation of glutamate homeostasis, leading to excitotoxicity via excessive receptor stimulation and calcium influx, is implicated in numerous neurological and neurodegenerative disorders, including PD^5^. This excitotoxic mechanism, often associated with altered glutamate transporter and receptor expression, contributes to neuronal cell death and is potentially influenced by inflammatory processes^6^. 

 The striatum, integral to motor control, receives glutamatergic inputs from the cortex and thalamus^7^. In PD, dopamine neuron loss disrupts striatal circuitry, potentially leading to excessive glutamatergic stimulation and motor impairment^8^. Neuroimaging and plasma studies suggest increased glutamatergic neurotransmission in PD^9,10^. The role of these glutamate alterations, whether causal or consequential to neurodegeneration, remains unclear. However, sustained glutamatergic excitation of the substantia nigra pars compacta neurons, potentially through disinhibition of the subthalamic nucleus, may contribute to further dopamine neuron loss^11^. 

 Glutamate-mediated excitotoxicity is central to neurodegenerative disease progression, making N-methyl-D-aspartate (NMDA) receptors a therapeutic target. While traditional NMDA antagonists induce significant side effects, limiting clinical utility, advances in the understanding of receptor subunit have enabled the development of more selective agents, such as negative allosteric modulators, with improved safety profiles. Current symptomatic treatments offer limited disease-modifying effects^12^. 

 Memantine and amantadine act as uncompetitive NMDA receptor antagonists, but they possess distinct pharmacological profiles. Memantine is a low-affinity, open-channel blocker that prevents chronic, excessive calcium influx associated with excitotoxicity while allowing normal physiological synaptic transmission to continue^13-15^. In contrast, amantadine acts as both an NMDA receptor antagonist and a dopaminergic agent, known to increase dopamine release and block dopamine reuptake, which may offer combined symptomatic and neuroprotective benefits^16^. 

 While glutamate-mediated excitotoxicity contributes to neurodegeneration in PD, the clinical efficacy and safety of NMDA receptor antagonists (e.g., memantine, amantadine) for managing debilitating NMS — particularly cognitive decline and neuropsychiatric disturbances — remain poorly defined. Therefore, this systematic review aims to synthesize current evidence on their therapeutic utility, addressing a critical gap in the comprehensive management of PD. 

## METHODS

### Eligibility criteria

 This review included studies involving participants diagnosed with idiopathic PD who received treatment with an NMDA receptor antagonist.

**Eligible studies featured:**
- A comparison group, such as a placebo, standard PD medication, or no intervention.
- Assessment of at least one of the following NMS: cognitive impairment, autonomic dysfunction, or depression.
- Randomized controlled trials and observational studies meeting predefined quality criteria.
- Only studies published in English or that could be translated.

**Conversely, studies were excluded if they:**
- Involved participants with secondary parkinsonism or other neurodegenerative diseases.
- Investigated nmda receptor antagonists for conditions other than pd.
- Compared different nmda receptor antagonists without a placebo or standard therapy group.
- Did not assess the specified nms.
- Were case reports, case series, expert opinions, studies with significant methodological limitations (e.g., inadequate randomization, high dropout rates, lack of blinding), and abstracts without full-text availability.

### Outcome

 Data extraction encompassed primary, secondary, and safety outcomes. Primary outcome measures included quantitative data for each intervention group, specifically focusing on scores obtained from cognitive assessments, measures of autonomic dysfunction, and depression scales. Secondary outcomes, as reported within each study, were also extracted. Furthermore, data on adverse events, including both incidence and severity, were extracted for all intervention and comparison groups to assess the tolerability and safety profiles of the NMDA receptor antagonists under investigation. A detailed description of the assessment scales utilized across the included studies is provided in Supplementary Material Table S1 (available at  https://www.demneuropsy.org/wp-content/uploads/2026/07/DN-2026.0496-Supplementary-Material.docx ). 

### Timepoints of interest

 The primary outcome measures were extracted at two predefined timepoints: 12 weeks and 24 weeks following the initiation of the intervention. These timepoints were chosen to capture both short-term and intermediate-term effects. Furthermore, to account for potential heterogeneity in reporting across the included studies, all reported outcome timepoints were systematically recorded and considered during the synthesis, allowing for a broader understanding of the temporal effects. 

### Report characteristics

 A systematic search was conducted for studies published in English from January 2000 to September 2025. Studies published in other languages were included if a validated English translation was accessible. No geographical restrictions were imposed. Both published and “in-press” articles were evaluated for eligibility. 

### Search methods

 This systematic review employed a comprehensive search strategy across major electronic databases, including PubMed and the Cochrane Library. 

 These databases were selected for their extensive coverage of biomedical literature, particularly regarding PD and its treatment. To broaden the search and mitigate publication bias, gray literature sources were explored, such as Google Scholar and clinical trial registries were examined, specifically ClinicalTrials.govClinicalTrials.gov. 

### Search strategy

 The search strategy utilized the following keywords: ‘Parkinson’s disease dementia,’ ‘NMDA receptor antagonists,’ and ‘non-motor symptoms,’ alongside related terms such as ‘cognitive deficits,’ ‘mood problems,’ and ‘autonomic symptoms.’ Boolean operators were used to combine search terms, and the search was restricted to publications from January 2000 to September 2025. 

### Data selection software

 This review utilized both EndNote 20 and Covidence software. EndNote 20 served as the primary repository for all identified citations and full-text articles. Covidence was employed for duplicate removal, title and abstract screening, quality assessment, and data extraction. 

### Merge of search results and identification of duplicates

 Duplicate citations were identified and removed using Covidence systematic review management software. Following automated deduplication, a manual review by Jan Arel Orlino was conducted to ensure accuracy. Any citations erroneously identified as duplicates were reinstated for subsequent title and abstract screening. 

### Screening of titles and abstracts

 Two independent reviewers performed study selection. In the initial screening phase, titles and abstracts were assessed for relevance. Each reviewer categorized the studies as ‘include,’ ‘exclude,’ or ‘uncertain.’ Any disagreements between reviewers (i.e., ‘include’ vs. ‘exclude’ or ‘include’ vs. ‘uncertain’) were resolved through discussion and consensus. 

### Full-text screening

 Following title and abstract screening, reviewers 1 and 2 independently assessed the full text of potentially eligible articles. Each reviewer determined inclusion or exclusion and provided detailed reasons for exclusion. Discrepancies were addressed through discussion and, where necessary, consensus was reached. 

### Quality assessment of risk of bias

 The risk of bias of all included randomized controlled trials was assessed using the Cochrane Risk of Bias tool (RoB 2), which is specifically intended for this type of study. Two independent reviewers applied the tool to each included study, evaluating the risk of bias across five key domains: bias arising from the randomization process, bias due to deviations from intended interventions, bias due to missing outcome data, bias in the measurement of the outcome, and bias in the selection of the reported result. For each domain, a series of signaling questions were answered to reach a judgment of “low risk of bias,” “some concerns,” or “high risk of bias.” Any reviewer disagreements were resolved through discussion and consensus. The final risk of bias judgments were reported in a summary table and a risk of bias graph to inform the synthesis of the results. 

## RESULTS

### Study selection

 The study selection process, as outlined in the Preferred Reporting Items for Systematic Reviews and Meta-Analyses (PRISMA) flow diagram (Figure 1)^17,18^, began with the identification of records from three databases. A total of 529 records were identified from Cochrane (118), Google Scholar (319), and PubMed (92). After removing 36 duplicate records, 493 records were screened. The screening process resulted in the exclusion of 460 records, leaving 33 reports for retrieval. Out of these, 9 reports could not be retrieved. The remaining 24 reports were assessed for eligibility, which led to the exclusion of 14 for various reasons, including different study design (7), different population (2), different intervention (1), different language (1), and study results not being reported (3). Ultimately, the review included 10 new studies. 

**Figure 1:**
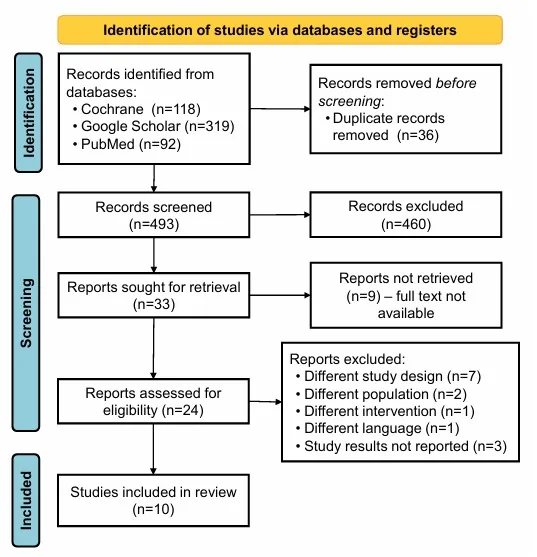
Preferred Reporting Items for Systematic Reviews and Meta-Analyses (PRISMA) flowchart.

Source: Generated using the PRISMA Flow Diagram Generator^18^.

### Study characteristics

 The included studies on NMDA antagonists for PD and related dementias varied in design, population, and duration. Most included studies were randomized, double-blind, placebo-controlled trials, considered the gold standard for clinical research. Some were also multicenter trials, which increased the generalizability of the findings. The study by Kawashima et al. was a unique pharmacological functional magnetic resonance imaging (fMRI) crossover protocol, and Litvinenko et al. conducted a non-randomized controlled study^13,19^. A few of the included studies were secondary or post-hoc analyses of larger, previously published trials (Table 1). 

**Table 1:** Study characteristics.

Study	Study Design & Population	Intervention & Duration	Key Findings & Measures
Kawashima et al.^13^	Crossover fMRI; N=10 (PD-MCI)	Memantine (titrated to 20 mg/ day) vs. Placebo 6 weeks	Worsened visuospatial working memory (2-back) and psychomotor speed (TMT-A); increased daytime sleepiness (ESS).
Emre et al.^20^	DB, placebo-controlled, parallel; N=199 (PDD or DLB)	Memantine (titrated to 20 mg/ day) vs. Placebo 24 weeks	Improved global clinical status (ADCS-CGIC) and behavioral symptoms (NPI) in DLB but not PDD. No significant difference in ADLs (ADCS-ADL23).
Aarsland et al.^21^	DB, placebo-controlled, parallel; N=72 (DLB or PDD)	Memantine vs. Placebo 24 weeks	Clinically relevant improvements in clinical status (ADCS-CGIC) with Memantine vs placebo.
Leroi et al.^14^	DB, placebo-controlled pilot; N=25 (PDD)	Memantine (20 mg/day) vs. Placebo 16 weeks	No significant benefit on primary cognitive scale (DRS); trend toward improved global functioning (CIBIC-Plus).
Larsson et al.^22^	Placebo-controlled RCT (secondary analysis); N=42 (DLB or PDD)	Memantine (titrated to 20 mg/ day) vs. Placebo 24 weeks	Significantly decreased severity of probable REM sleep behavior disorder (SSQ); no effect on daytime sleepiness (ESS).
Wesnes et al.^23^	DB, placebo-controlled RCT; N=51 (mild/moderate PDD or DLB)	Memantine (titrated to 20 mg/ day) vs. Placebo 24 weeks	Statistically significant improvements in attention and episodic memory (CDR System).
Litvinenko et al.^19^	Non-randomized controlled; N=62 (PD with dementia)	Memantine (20 mg/day) vs. Control (standard care) 52 weeks	Improved cognitive function (MMSE, ADAS-cog, FAB) and behavioral symptoms (NPI-12); slowed long-term progression.
Leroi et al.^15^	DB, placebo-controlled RCT (secondary analysis); N=25 (PDD)	Memantine (20 mg/day) vs. Placebo 16 weeks	Improved goal attainment (GAS) and significantly reduced caregiver burden (ZBI); no difference in patient quality of life (PDQ-8).
Ondo et al.^24^	DB, placebo-controlled pilot; N=40 (PD without dementia)	Memantine (titrated to 20 mg/ day) vs. Placebo 8 weeks	No significant improvements in non-motor assessments (ESS, FSS, Hamilton), though clinical trends favored memantine.
Mehta et al.^16^	Post-hoc analysis of Phase 3 trials; N=196 (PD with dyskinesia)	Amantadine ER (Gocovri) vs. Placebo 12 weeks	Improved daytime sleepiness and depressed mood (MDS-UPDRS Part I); significantly worsened cognitive impairment and hallucinations.

Abbreviations: 2-back, Visuospatial N-back test; ADAS-cog, Alzheimer’s Disease Assessment Scale-Cognitive Subscale; ADCS-ADL23, Alzheimer’s Disease Cooperative Study-Activities of Daily Living, 23-item; ADCS-CGIC, Alzheimer’s Disease Cooperative Study-Clinical Global Impression of Change; CDR System, Cognitive Drug Research Computerized Assessment System; CIBIC-Plus, Clinician’s Interview-Based Impression of Change Plus Caregiver Input; DB, Double-blind; DLB, Dementia with Lewy Bodies; DRS, Dementia Rating Scale; ER, Extended Release; ESS, Epworth Sleepiness Scale; FAB, Frontal Assessment Battery; FSS, Fatigue Severity Scale; GAS, Goal Attainment Scaling; MDS-UPDRS, Movement Disorder Society-Unified Parkinson’s Disease Rating Scale; MMSE, Mini-Mental State Examination; NPI/NPI-12, Neuropsychiatric Inventory; PDD, Parkinson’s Disease Dementia; PD-MCI, Parkinson’s Disease with Mild Cognitive Impairment; PDQ-8, Parkinson’s Disease Questionnaire-8; RCT, Randomized Controlled Trial; SSQ, Stavanger Sleep Questionnaire; TMT-A, Trail-Making Test Part A; ZBI, Zarit Burden Inventory.

Source: The authors.

 The sample sizes ranged from small pilot studies with 10–25 patients to larger trials with nearly 200 participants. Study durations also varied significantly, from an 8-week pilot trial to a long-term, 52-week study. The majority of studies lasted for either 16 or 24 weeks. 

 Clinical criteria carefully defined the patient populations. Most studies focused on patients with Parkinson’s disease dementia (PDD) and dementia with Lewy bodies (DLB). A few studies included patients with mild cognitive impairment (PD-MCI) or PD patients without dementia. Common inclusion criteria included specific scores on cognitive assessments like the Mini-Mental State Examination (MMSE) and a diagnosis based on established criteria like the UK Parkinson’s Disease Brain Bank Criteria. Exclusion criteria often included other forms of dementia, severe systemic diseases, or recent use of other relevant medications. 

### Quality assessment of risk of bias

 The quality of the studies included was assessed using the RoB 2. Two independent reviewers evaluated the risk of bias across five domains: randomization, deviations from intended interventions, missing outcome data, outcome measurement, and selection of the reported result. The final judgment for each study’s outcome was categorized as “low risk,” “some concerns,” or “high risk”. Any disagreements between the reviewers were resolved through discussion. 

 The assessment revealed significant heterogeneity in the methodological quality of the included studies. Only one study was considered to have a low risk of bias across all domains, owing to its robust design, high completion rate (96%), and transparent reporting^14^. 

 Three studies were categorized as having some concerns. The randomized crossover study raised concerns due to a high withdrawal rate (16.7%), which introduced a risk of bias related to missing outcome data^13^. Two studies were judged to have some concerns because they were secondary analyses or lacked a predefined primary endpoint, raising the possibility of selective reporting^14,20^. 

 Most studies (n=5) were judged to have a high risk of bias, which significantly limits the reliability of their findings. One study was a non-randomized controlled trial, a design that inherently fails to protect against confounding and selection bias^19^. Four other studies were either exploratory pilot trials or secondary/posthoc analyses of data, where the lack of a predefined primary outcome and statistical adjustments for multiple comparisons introduced a significant risk of selective reporting bias^21-24^. Similarly, the post-hoc analysis was also assigned a high risk of bias due to the potential for selective reporting^16^. 

 These findings are synthesized and presented in a summary table and a risk of bias graph to inform the interpretation of the results (Figures 2 and 3)^25^. 

**Figure 2:**
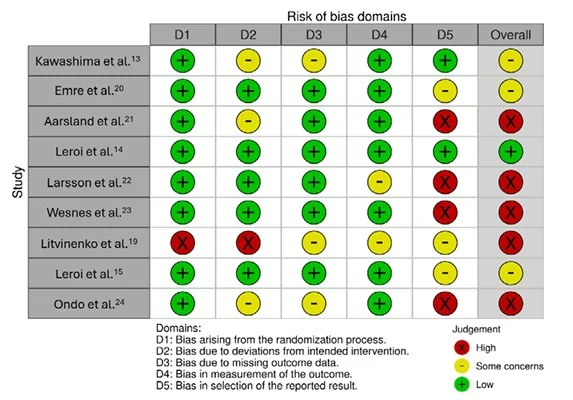
Summary table for the risk of bias of extracted studies.

Source: Generated using the robvis tool^18^.

**Figure 3:**
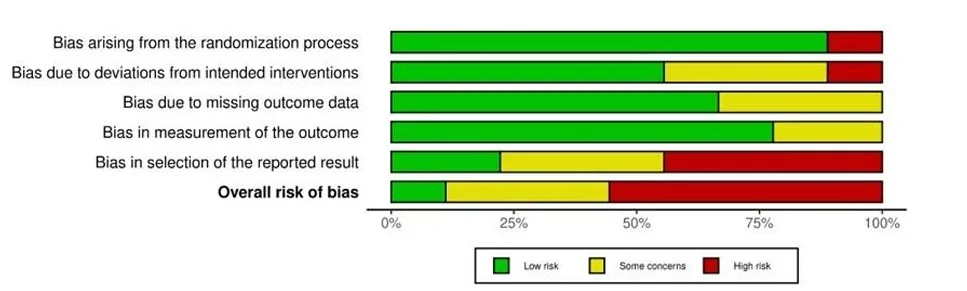
Risk of bias graph.

Source: Generated using the robvis tool^25^.

### Key findings of outcomes

#### Efficacy of memantine and amantadine in neuropsychiatric symptoms

##### Memantine

 The effectiveness of memantine in treating cognitive impairment and NMS in patients with PD, PD-MCI, PDD, and DLB is mixed. Several studies reported positive effects on primary cognitive outcomes, including improvements in choice reaction time, word recognition, and various cognitive test scores (e.g., MMSE, Alzheimer’s Disease Assessment Scale-Cognitive Subscale [ADAS-cog])^19,20,21,23^. Some studies also noted a benefit on secondary outcomes, such as behavioral symptoms including disinhibition, irritability, and hallucinations^19^, and a reduction in caregiver burden^14^. 

 In contrast, other studies found no significant benefit or even detrimental effects. A study found that memantine worsened visuospatial working memory and slowed cognitive processing^13^. Another study reported no statistically significant benefit on the primary outcome measure, the Dementia Rating Scale (DRS), although a trend toward improvement in global functioning was observed^14^. The effects of memantine on autonomic dysfunction also appeared to be varied, with one study showing a positive effect on probable REM sleep behavior disorder^22^, while another found no significant improvements in non-motor assessments^24^. 

 A secondary analysis showed that memantine significantly reduced caregiver burden (Zarit Burden Inventory) and improved Goal Attainment Scaling outcomes.^15^

##### Amantadine

 The findings for amantadine extended-release (Gocovri) are also mixed. A post-hoc analysis indicated that it significantly improved daytime sleepiness and depressed mood, suggesting a benefit for some NMS^16^. 

### Safety and tolerability

 The review of adverse events indicates distinct safety profiles for the two interventions. While memantine was associated with a trend for increased daytime sleepiness, amantadine extended-release was linked to more severe neuropsychiatric side effects^13^. Specifically, the study on Gocovri reported a significant worsening of both investigator-rated cognitive impairment and hallucinations/psychosis compared to placebo, highlighting a potential trade-off between the benefits for mood/sleep and adverse cognitive effects^16^. The high rate of patients choosing to continue memantine after one study’s conclusion suggests a perception of clinical benefit and acceptable tolerability, although specific adverse events were not detailed in that finding^24^. 

## DISCUSSION

 This systematic review analyzed the efficacy and safety of NMDA receptor antagonists for NMS in patients with PD. The primary outcomes of interest were changes in scores on cognitive assessments, measures of autonomic dysfunction, and depression scales, while secondary outcomes included other reported effects and a comprehensive review of adverse events. 

### Efficacy of N-methyl-D-aspartate antagonists on non-motor symptoms

 While PDD and DLB share overlapping neuropathology and clinical features, their therapeutic management in the broader literature often differs^26^. First-line treatment for cognitive impairment in both conditions typically involves cholinesterase inhibitors^27^. 

 However, patients with DLB usually present with cognitive decline and neuropsychiatric symptoms (e.g., severe visual hallucinations) earlier in the disease course and exhibit a profound sensitivity to neuroleptic medications^26,28^. Furthermore, standard dopaminergic therapies used to treat motor symptoms in PDD are often poorly tolerated in DLB, as they can rapidly exacerbate psychosis^28^. 

### Cognitive function

 The review found mixed results for NMDA antagonists on cognitive function. Memantine’s effectiveness seems to vary depending on the patient’s level of cognitive impairment. Its impact is more noticeable in individuals with PDD or DLB, rather than in those with MCI or without NMS. 

 In patients with established PDD or DLB, memantine significantly improved measures in attention and episodic memory (choice reaction time, immediate and delayed word recognition). These improvements were found in patients with PDD and DLB. The effect sizes were considered clinically relevant^21,23^. 

 Conversely, in a small pilot study of patients with MCI in PD (PD-MCI), memantine unexpectedly worsened performance on a visuospatial working memory test. It increased the time needed to complete the psychomotor speed and attention task (the 2-back test and the Trail-Making Test Part A)^13^. 

 The cognitive effectiveness of memantine remains mixed; it did not improve global cognitive measures (DRS, MMSE) in one PDD trial, but did show significant improvement on the MMSE, ADAS-cog, Frontal Assessment Battery (FAB), and clock drawing test in another^14,19^. 

 The extended-release form of amantadine was found to significantly worsen investigator-rated cognitive impairment and hallucinations. 

### Neuropsychiatric symptoms

 For other neuropsychiatric manifestations, memantine showed a significant benefit for certain behavioral symptoms, including a reduction in agitation, aggression, irritability, anxiety, and hallucinations. 

 A large trial found that memantine significantly improved Neuropsychiatric Inventory (NPI) scores for agitation/aggression and appetite/eating disorder in the total patient population. It showed significant benefits in the DLB subgroup for the total NPI score and for delusions, hallucinations, and sleep^20^. 

 The post-hoc analysis of extended-release amantadine indicated that it significantly improved daytime sleepiness and depressed mood^16^. 

### Sleep

 Two studies specifically examined sleep-related NMS. Memantine was found to significantly decrease probable REM sleep behavior disorder symptoms in patients with DLB and PDD^22^. 

 However, its effect on excessive daytime sleepiness was mixed. One study found no significant effect on the Epworth Sleepiness Scale (ESS), while another found a trend toward higher scores with memantine. A post-hoc analysis of amantadine, a related drug, significantly improved daytime sleepiness^13^. 

### Trends and other findings

 Several trends and findings emerged from studies. Deterioration upon discontinuation of medication is a concern, with a study indicating that more participants on memantine experienced global deterioration after stopping the drug compared to those on a placebo^14^. A recurring limitation across many studies was the small sample sizes. Furthermore, there is a suggestion that patient-reported outcomes, specifically clinimetric measures like Goal Attainment Scaling, might be more effective than conventional measures for detecting improvements in PDD, as proposed by a study^14^. 

### Study limitations

 While the review found evidence that NMDA receptor antagonists can benefit certain NMS in PD, the findings are limited by the significant heterogeneity and high risk of bias across the included studies. Only one study was considered to have a low risk of bias^14^. Most studies were either exploratory pilot trials, non-randomized, or post-hoc analyses, which introduced a significant risk of selective reporting and other biases. These methodological limitations underscore the need for more robust, high-quality, randomized controlled trials to definitively determine the clinical utility of these drugs for the NMS in PD. 

### Future recommendations

 Based on the limitations identified in this systematic review, future research should prioritize the following recommendations to address the current knowledge gaps: 

 Conduct more high-quality randomized controlled trials: The review found that most included studies had a high risk of bias, with only one study rated as having a low risk. Future studies should follow rigorous randomized controlled trial designs with adequate randomization, blinding, and predefined primary endpoints to minimize bias and improve the reliability of findings. 

 Standardize outcome measures: The review highlighted various cognitive, psychiatric, and sleep assessment measures. Future studies should strive for a more standardized set of validated scales and tests for better comparison and meta-analysis of results across different trials. 

 Investigate specific patient populations: The review found that memantine’s efficacy may depend on the degree of cognitive impairment, showing different effects in patients with PD-MCI versus those with PDD/DLB. Future research should focus on a more nuanced investigation of specific patient subgroups to determine which NMDA antagonists are most effective for different stages and types of NMS. 

 Explore novel NMDA antagonists: While this review focused on memantine and amantadine, future studies should investigate new, more selective NMDA receptor antagonists with improved safety profiles to potentially avoid the side effects seen with the current generation of drugs. 

 In conclusion, this systematic review synthesized the current evidence on the efficacy and safety of NMDA receptor antagonists for the NMS in PD. The findings reveal that the effectiveness of these treatments is varied and often dependent on the specific drug, the patient population, and the type of NMS being treated. 

 The evidence for memantine, the most studied drug, is inconsistent. While some studies showed a benefit for cognitive and behavioral symptoms in patients with established PDD and DLB, others reported no significant improvement or even worsening of cognitive function in patients with MCI. For other NMS, memantine showed a significant benefit for certain behavioral symptoms, including a reduction in agitation, aggression, irritability, anxiety, and hallucinations. It also decreased symptoms of probable REM sleep behavior disorder. Amantadine extended-release showed promise for improving daytime sleepiness and depressed mood but was associated with a significant worsening of cognitive impairment and psychosis. 

 Ultimately, the results of this review are significantly limited by the methodological heterogeneity and high risk of bias across the included studies. Only one of the studies evaluated was found to have a low risk of bias, meaning the findings from the others are less reliable. These limitations highlight a critical need for more rigorous, high-quality randomized controlled trials to establish the true clinical utility of NMDA antagonists for treating the NMS in PD. Future research should also focus on standardizing outcome measures and investigating specific patient subgroups to better understand who might benefit most from these treatments. 

## Data Availability

The datasets generated and/or analyzed during the current study are available from the corresponding author upon reasonable request.
