# Supplementary material for: The efficacy of NMDA receptor antagonists in neuropsychiatric and non-motor symptoms of Parkinson’s disease: a systematic review
Source: Dement Neuropsychol. 2026 Nov 2;20:e20260496. doi: 10.1590/1980-5764-DN-2026-0496 (PMC13733341; doi:10.1590/1980-5764-DN-2026-0496)
Supplement: Supplementary file 1 [file 1980-5764-dn-20-e20260496-md01.docx]

**Supplementary Material**

**Table S1.** Scales for assessment

| Scale Name | Description | References |
| --- | --- | --- |
| Unified Parkinson's Disease Rating Scale (UPDRS) | A scale used to assess the severity of motor symptoms in Parkinson's disease. Part III (motor section) is specifically mentioned. | 13, 14, 15, 18, 19, 23 |
| Epworth Sleepiness Scale (ESS) | An eight-item scale used to measure excessive daytime somnolence, rated by a carer with scores ranging from 0-24, with higher scores indicating abnormal sleepiness. | 13, 21 |
| Mini-Mental State Examination (MMSE) | A scale used to assess global cognitive function. Scores range from 10-24 in one study, 12 and higher in others, and 10-27 in another. | 13, 14, 15, 18, 19, 23 |
| Montreal Cognitive Assessment (MOCA) | A scale used to assess global cognitive function. | 13 |
| Trail-Making Test Parts A & B (TMT-A & TMT-B) | TMT-A assesses psychomotor speed and attention, while TMT-B assesses executive function and rapid set shifting. | 13, 19 |
| Alzheimer's Disease Cooperative Study (ADCS)-Clinical Global Impression of Change (ADCS-CGIC) | A scale used to assess global clinical status, with scores from 1 (substantial improvement) to 7 (substantial worsening) and 4 indicating no change. It is assessed by an experienced clinician. | 19, 22 |
| Alzheimer's Disease Cooperative Study-Activities of Daily Living, 23-item (ADCS-ADL23) | A 23-item scale with scores from 0 to 78, where a higher score indicates better performance in activities of daily living. | 19 |
| Zarit Burden Inventory (ZBI) | A 22-item scale that measures subjective caregiver burden with scores from 0 (best state) to 4 (worst state) for each item. Higher total scores indicate greater burden. | 15, 19 |
| Neuropsychiatric Inventory (NPI) / NPI-12 | A 12-item scale with a maximum score of 144, where higher scores indicate worse behavioral symptoms. It assesses psychiatric symptoms and behavioral impairments. | 14, 18, 19, 22 |
| Dementia Rating Scale (DRS) | A scale used to assess global cognitive impairment with an emphasis on subcortical functions. | 14, 15 |
| Clinician's Interview-Based Impression of Change Plus Caregiver Input (CIBIC-Plus) | A tool used to assess global change by an independent assessor. | 14 |
| CDR System Tests | An integrated set of automated tests of attention and memory administered via a computer with a response box. It includes tests for Simple Reaction Time (SRT), Choice Reaction Time (CRT), and Word Recognition (Immediate and Delayed). | 20, 22 |
| Stavanger Sleep Questionnaire (SSQ) | A questionnaire rated by a carer in an interview with a clinician to evaluate sleep disturbances. It includes a question on probable REM sleep behavior disorder. | 21 |
| Disability Assessment Dementia (DAD) scale | A scale used to assess disability and self-care ability. | 18 |
| Frontal lobe dysfunction Assessment Battery (FAB) | A scale used to assess frontal lobe dysfunction. | 18 |
| Goal Attainment Scaling (GAS) | A patient-reported outcome measure where individually determined goals are scored from -2 (worse than expected) to +2 (much better than expected). A score of 50 indicates no change, and higher scores mean greater improvement. | 15 |
| Parkinson's Disease Questionnaire-8 (PDQ-8) | A scale that assesses health-related Quality of Life (QoL), with a summary index score of 0-100, where higher scores indicate worse disability. | 15 |
| Movement Disorder Society-Unified Parkinson's Disease Rating Scale (MDS-UPDRS) Part I | A 13-item scale that assesses non-motor aspects of daily living. It includes investigator-rated items like cognition and hallucinations, and patient-rated items such as sleep problems and fatigue. | 16 |
